# Supplementary material for: Most mitochondrial dGTP is tightly bound to respiratory complex I through the NDUFA10 subunit
Source: Commun Biol. 2022 Jun 23;5:620. doi: 10.1038/s42003-022-03568-6 (PMC9226000; doi:10.1038/s42003-022-03568-6)

Most mitochondrial dGTP is tightly bound to respiratory complex I through the NDUFA10 subunit

David Molina-Granada[1,2], Emiliano González-Vioque[1,2&], Marris G. Dibley[3], Raquel Cabrera-Pérez[1,2], Antoni Vallbona-Garcia[1], Javier Torres-Torronteras[1,2], Leonid A. Sazanov[4], Michael T. Ryan[3], Yolanda Cámara[1,2,†,*] and Ramon Martí[1,2,†,*]

[1] Research Group on Neuromuscular and Mitochondrial Disorders, Vall d'Hebron Institut de Recerca, Universitat Autònoma de Barcelona, Barcelona, Catalonia
[2] Biomedical Network Research Centre on Rare Diseases (CIBERER), Instituto de Salud Carlos III, Madrid, Spain
[3] Department of Biochemistry and Molecular Biology, Monash Biomedicine Discovery Institute, Monash University, Melbourne, Australia
[4] Institute of Science and Technology Austria, Klosterneuburg, Austria.
[&] Current address: Department of Clinical Biochemistry. Hospital Universitario Puerta del Hierro-Majadahonda

†These authors contributed equally to this work.
*Corresponding authors
E-mail: ramon.martí@vhir.org (RM)
yolanda.camara@vhir.org (YC)

LIST OF SUPPLEMENTARY TABLES

Supplementary Table 1: dGTP-interacting proteins identified by affinity chromatography

Supplementary Table 2: List of antibodies used in the study

Supplementary Table 3: List of materials used for immunoprecipitation and dNTP pull-down assays

Supplementary Table 4: Oligonucleotides used for dNTP concentration determination

Supplementary Table 5: List of radiochemicals used in the study

Supplementary Table 6: List of primers and probes used for mtDNA copy number quantification by Real-time PCR

Supplementary Table 7: List of relevant materials used in the study

LIST OF SUPPLEMENTARY FIGURES

Supplementary Figure 1: Percent of dNTP recovery in mitochondrial extracts prepared with different methods.

Supplementary Figure 2: Affinity chromatography with immobilized ɣ-amino-octyl-dGTP

Supplementary Figure 3: Mutations introduced in NDUFA10 deoxyribonucleoside kinase (dNK) domain to disable dGTP binding

Supplementary Figure 4: Co-migration analysis of dGTP with complex I in sucrose-density gradients with different detergents

Supplementary Figure 5: Seahorse oxygen consumption assay of NDUFA10 mutant cell lines.

Supplementary Figure 6: MtDNA copy number and deletion analysis in NDUFA10^{KO} and NDFUA10^{KI} cells

Supplementary Figure 7: Uncropped western blot images for the indicated figures

Band	Accession	Protein	Nucleotide-binding activcity	MW [kDa]	Scores	#Peptides	SC [%]	# Spec Counts
1	TGM2_MOUSE	Protein-glutamine gamma-glutamyltransferase 2 OS=Mus musculus GN=Tgm2 PE=1 SV=4	GTP- binding protein	77	4254.3	32	38.3	178
	NDUS1_MOUSE	NADH-ubiquinone oxidoreductase 75 kDa subunit, mitochondrial OS=Mus musculus GN=Ndufs1 PE=1 SV=2		79.7	3754.3	46	38.7	155
	GRP78_MOUSE	78 kDa glucose-regulated protein OS=Mus musculus GN=Hspa5 PE=1 SV=3	ATP-binding protein	72.4	745.3	17	24.9	27
	FINC_MOUSE	Fibronectin OS=Mus musculus GN=Fn1 PE=1 SV=3		272.3	667.9	17	6.6	26
	ECHA_MOUSE	Trifunctional enzyme subunit alpha, mitochondrial OS=Mus musculus GN=Hadha PE=1 SV=1		82.6	644.6	19	19.9	27
2	NDUAA_MOUSE	NADH dehydrogenase [ubiquinone] 1 alpha subcomplex subunit 10, mitochondrial OS=Mus musculus GN=Ndufa10 PE=1 SV=1	Complex I subunit	40.6	6762.3	26	41.7	287
	ACTB_MOUSE	Actin, cytoplasmic 1 OS=Mus musculus GN=Actb PE=1 SV=1		41.7	1176.3	22	32.3	41
	H2AY_MOUSE	Core histone macro-H2A.1 OS=Mus musculus GN=H2afy PE=1 SV=3		39.7	707.5	11	26.1	23
	ACTBL_MOUSE	Beta-actin-like protein 2 OS=Mus musculus GN=Actbl2 PE=1 SV=1		42	297.5	10	14.1	16
	H4_MOUSE	Histone H4 OS=Mus musculus GN=Hist1h4a PE=1 SV=2		11.4	278.6	6	40.8	10
3	DGUOK_MOUSE	Deoxyguanosine kinase, mitochondrial OS=Mus musculus GN=Dguok PE=2 SV=2	Deoxyucleoside salvage kinase	32.2	2107.4	12	21.3	91
	NDUV2_MOUSE	NADH dehydrogenase [ubiquinone] flavoprotein 2, mitochondrial OS=Mus musculus GN=Ndufv2 PE=1 SV=2		27.3	637.1	8	25.8	30
	RAB5C_MOUSE	Ras-related protein Rab-5C OS=Mus musculus GN=Rab5c PE=1 SV=2	GTP- binding protein	23.4	425	8	32.9	13
	RAB21_MOUSE	Ras-related protein Rab-21 OS=Mus musculus GN=Rab21 PE=1 SV=4	GTP- binding protein	24.1	414.7	9	25.7	13
	RAB5B_MOUSE	Ras-related protein Rab-5B OS=Mus musculus GN=Rab5b PE=1 SV=1	GTP- binding protein	23.7	308.2	6	30.2	10
4	NDUA7_MOUSE	NADH dehydrogenase [ubiquinone] 1 alpha subcomplex subunit 7 OS=Mus musculus GN=Ndufa7 PE=1 SV=3		12.6	988.4	16	58.4	36
	H2B1C_MOUSE	Histone H2B type 1-C/E/G OS=Mus musculus GN=Hist1h2bc PE=1 SV=3		13.9	703.5	10	66.7	31
	H2B2B_MOUSE	Histone H2B type 2-B OS=Mus musculus GN=Hist2h2bb PE=1 SV=3		13.9	638.2	10	66.7	28
	H2B1B_MOUSE	Histone H2B type 1-B OS=Mus musculus GN=Hist1h2bb PE=1 SV=3		13.9	620.1	10	66.7	28
	NDUB4_MOUSE	NADH dehydrogenase [ubiquinone] 1 beta subcomplex subunit 4 OS=Mus musculus GN=Ndufb4 PE=1 SV=3		15.1	359.6	6	34.9	14

Supplementary Table 1: dGTP-interacting proteins identified by affinity chromatography. Pulled-down proteins by affinity chromatography with immobilized ɣ-amino-octyl-dGTP and control blank agarose were resolved by SDS-PAGE and stained with Coomassie Blue G250. Four bands were visualized exclusively in the immobilized ɣ-amino-octyl-dGTP pull-down (Bands 1-4 in Supplementary figure 2). The table shows a curated list of the majority identified peptides by MASCOT analysis for each area. Molecular weight (MW), number of identified peptides (#peptides), Mascot score, sequence coverage (%[sc]), and number of spec counts for each protein are also included in the table. Ribonucleotide binding activity is indicated when previously reported for a protein in the table. Components of mitochondrial complex I are shaded in grey. Majority proteins are shadowed in black.

Antibodies for western-blot	Company	Catalogue #	usual working dilution
polyclonal rabbit anti mouse Ig HRP	Dako	P0280	1:5000
polyclonal goat anti-rabbit Ig HRP	Dako	P0448	1:5000
Anti-NDUFA10 antibody	GeneTex	GTX114572	1:1000
Anti-Core II Complex III	Molecular Probes	A11143	1:1000
Anti-COX IV	Abcam	ab16056	1:1000
Anti-SDHA70	Abcam	ab14715	1:2000
Anti-VDAC	Abcam	ab15895	1:5000
Anti-TFAM	GeneTex	GTX103231	1:1000
Anti-39kDa subunit Complex I	Molecular Probes	A21344	1:1000
Anti-FLAG M2 antibody	Sigma-Aldrich	F3165	1:1000
Anti-GAPDH	Origene	TA802519	1:1000

Supplementary Table 2: List of antibodies used in the study

Inmunoprecipitation and pull-down	Company	Catalogue #
Complex I Immunocapture Kit beads	Abcam	ab109711
Immobilized Protein G Agarose beads	Abcam	ab174816
Mouse IgG Agarose	Sigma-Aldrich	A0919
ANTI-FLAG M2 Magnetic BEADS	Sigma-Aldrich	M8823
3xFLAG peptide	Sigma-Aldrich	F4799
immobilized ɣ-amino-octyl-dCTP	Jena Bioscience	AC-108L
immobilized ɣ-amino-octyl-dGTP	Jena Bioscience	AC-112L
blank agarose	Jena Bioscience	AC-001L

Supplementary Table 3: List of materials used for immunoprecipitation and dNTP pull-down assays

Oligonucleotides for dNTP quantification	
dNTPenz	5'-CCGCCTCCACCGCC-3'
dATPenz	5'-AAATAAATAAATAAATAAATGGCGGTGGAGGCGG-3'
dTTPenz	5'-TTATTATTATTATTATTAGGCGGTGGAGGCGG-3'
dCTPenz	5'-TTTGTTTGTTTGTTTGTTTGGGCGGTGGAGGCGG-3'
dGTPenz	5'-TTTCTTTCTTTCTTTCTTTCGGCGGTGGAGGCGG-3'

Supplementary Table 4: Oligonucleotides used for dNTP concentration determination by a radiometric polymerase-based assay. dNTPenz acts as the common short primer that is annealed with the different longer templates for the specific determination of all four dNTPs,

Radiochemicals	Company	Catalogue #
2’-deoxyguanosine, [8-^{3}H]	Vitrax	VT120
[^{3}H]-dATP	Moravek	MT644
[^{3}H]-dTTP	PerkinElmer	NET221X001MC
[α-^{32}P]-dGTP EasyTide	PerkinElmer	NEG514H250UC
[α-^{32}P]-dCTP EasyTide	PerkinElmer	NEG513H100UC
[α-^{32}P]-dATP EasyTide	PerkinElmer	NEG012H250UC
[α-^{32}P]-dTTP EasyTide	PerkinElmer	NEG505H250UC

Supplementary Table 5: List of radiochemicals used in the study

**mtDNA:**
Primer 12SrRNA-Fw: 5'- CCA CGG GAA ACA GCA GTG AT -3' (ntd 805-824*)<br>Primer 12SrRNA-Rv: 5'- CTA TTG ACT TGG GTT AAT CGT GTG A -3' (ntd 903-927*)<br>probe 12SrRNA: FAM -5'- TGC CAG CCA CCG CG -3'- MGB (ntd 888-900*)<br><br>* RefSeq. NC_012920<br>Standard: nucleotides 805-927 (RefSeq. NC_012920.1) cloned in pcR2.1_TOPO vector (Invitrogen)
**nDNA:**
RNAsaP Control Reagent Taqman assay: Ref 4316844 ThermoFisher Scientific (VIC-TAMRA)<br>Standard: nucleotides 4707-4793 (RefSeq. NG_033959.1) cloned in pcR2.1_TOPO vector (Invitrogen)

Supplementary Table 6: List of primers and probes used for mtDNA copy number quantification by real-time PCR

List of relevant materials	Company	Catalogue #
1,1, 2-trichlorotrifluoroethane	Fluka	91440
Acrylamide:bisacrylamide solution (37.5:1)	Bio-Rad	1610148
Antimycin A	Sigma-Aldrich	A8674
Bovine serum albumin, essentially fatty acids free	Sigma-Aldrich	A6003
Carbonyl cyanide m-chlorophenyl hydrazone (CCCP)	Sigma-Aldrich	C2759
cOmplete EDTA-free protease inhibitor cocktail	Roche	11873580001
Coomassie Blue G250	Sigma-Aldrich	B0770
Decilubiquinone	Sigma-Aldrich	D7911
Diethylaminoethyl (DEAE) filtermat filters	PerkinElmer	1450-522
Dulbecco's Modified Eagle Medium (DMEM) high-glucose with GlutaMAX	Thermo Fisher Scientific	31966021
IGEPAL CA-630	Sigma-Aldrich	I8896
Immobilon Western Chemiluminescent HRP Substrate (ECL)	Millipore	WBKLS0500
Immun-Blot PVDF Membranes for Protein Blotting	Bio-Rad	1620177
LA Taq polymerase	Takara	R002A
NADH	Sigma-Aldrich	N8129
n-dodecyl β-D-maltoside (DDM)	anatrace	D310
Oligomycin A	Sigma-Aldrich	75351
PEI 25K transfection reagent	Polysciences Inc.	23966
Penicilin/Streptomycin	Cultek	H3DE17-602E
Perchloric acid (PCA)	Merck	1005141000
Phosphatase inhibitor cocktail PhosSTOP	Roche	4906837001
Pierce Comassie Bradford Protein Assay	Thermo Fisher Scientific	10270014
PlusOne Silver Staining Kit, Protein	GE Healthcare	17-1150-01
Poly-D-lysine	Sigma-Aldrich	P7405
Puromycin	Gibco	A11138
Rotenone	Sigma-Aldrich	R8875
TaqMan Universal Master Mix II, with UNG	Applied Biosystems	4440046
Termosequenase	GE Healthcare	E79000Y
Trichloracetic acid (TCA)	Sigma-Aldrich	T0699
Trioctylamine	Sigma-Aldrich	T81000
Triton X-100	Sigma-Aldrich	X-100
Ultima Gold scintillation liquid cocktail	PerkinElmer	6013329
Whatman DE81 filters	GE Healthcare	3658-023

Supplementary Table 7: List of relevant materials used in the study

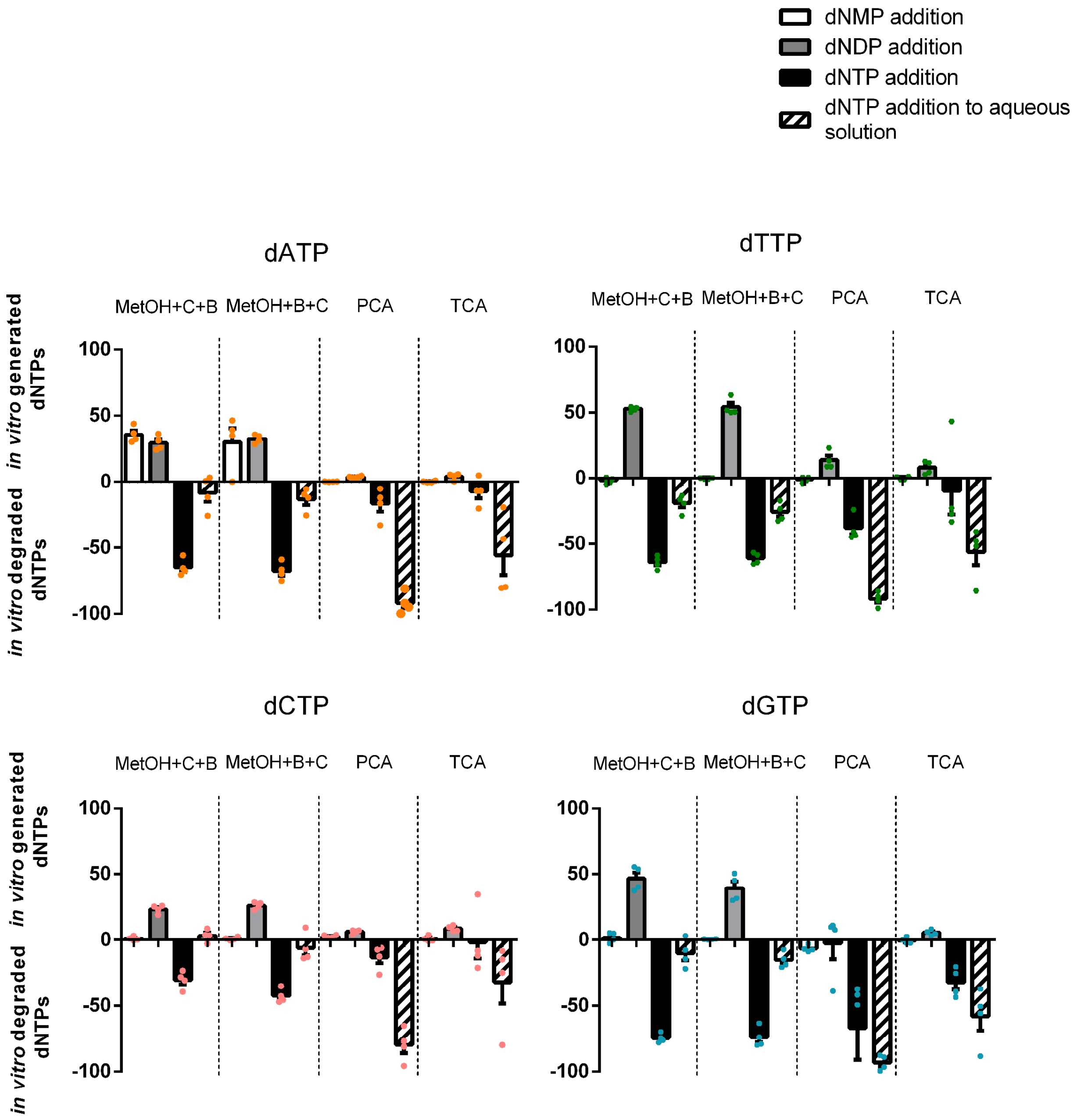

Supplementary Figure 1: Percent of dNTP recovery in mitochondrial extracts prepared with different methods. 100 pmol of the four canonical dNMPs, dNDPs, or dNTPs were added to liver mitochondrial pellets immediately after resuspension in 0.5 M TCA, 0.6 M PCA, or 60% ice-cold methanol. Extraction was performed using standard methods (TCA, PCA, MetOH+C+B (methanol incubation, plus centrifugation followed by boiling of supernatants) or MetOH+B+C (methanol incubation, boiling and later centrifugation of samples)) and dATP, dTTP, dCTP, and dGTP concentration was determined. Percent of dNTP recovery of the expected value was calculated, considering the concentration in non-added samples (the red line marks 100% recovery). Deviations from 100% indicate the presence of residual enzyme activities or chemical degradation that alter the final dNTP pool during the extraction procedure. Values above 100% indicate added dNMPs; dNDPs are converted to dNTPs. Values below 100% suggest dNTP degradation. Results are mean values from 4 independent experiments (symbols). Error bars indicate SEM.

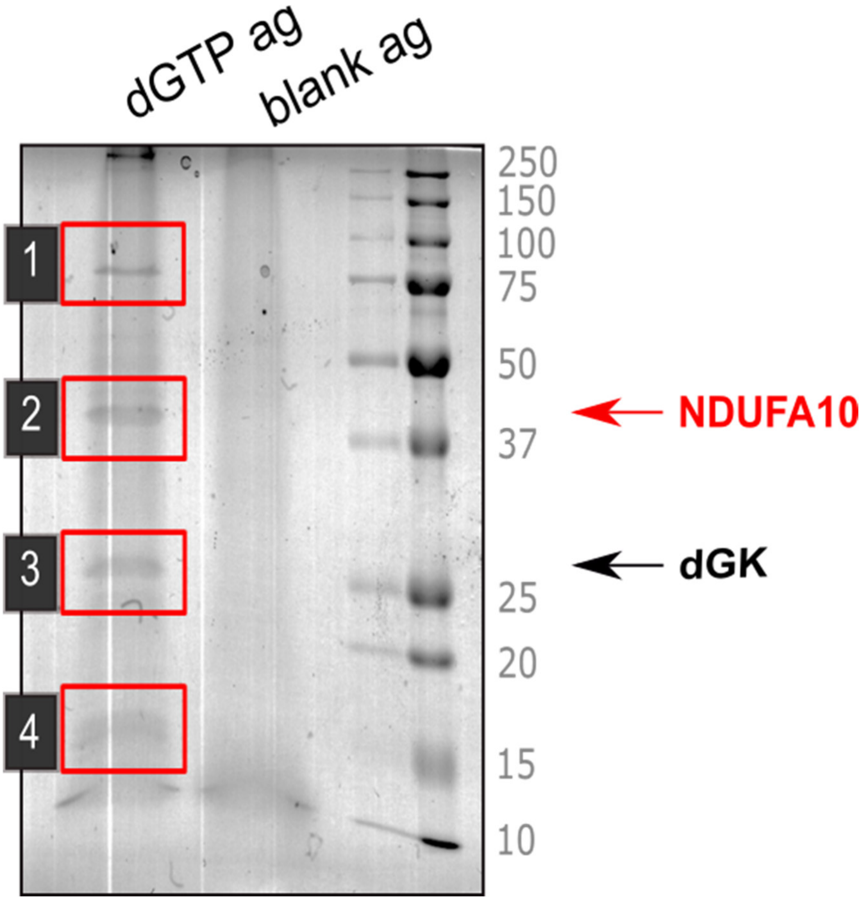

Supplementary Figure 2: Affinity chromatography with immobilized ɣ-amino-octyl-dGTP (dGTP ag). Pulled-down proteins from a native liver mitochondrial extract were resolved in 12% SDS-PAGE and stained with Coomassie blue G250. A pull-down with blank agarose was run in parallel as a control for unspecific binding. We selected four areas (1-4 in the image) and trypsin digest for further peptide analysis by Orbitrap LC-MS/MS. A curated list of identified peptides for each selected band is shown in Supplementary Table 1.

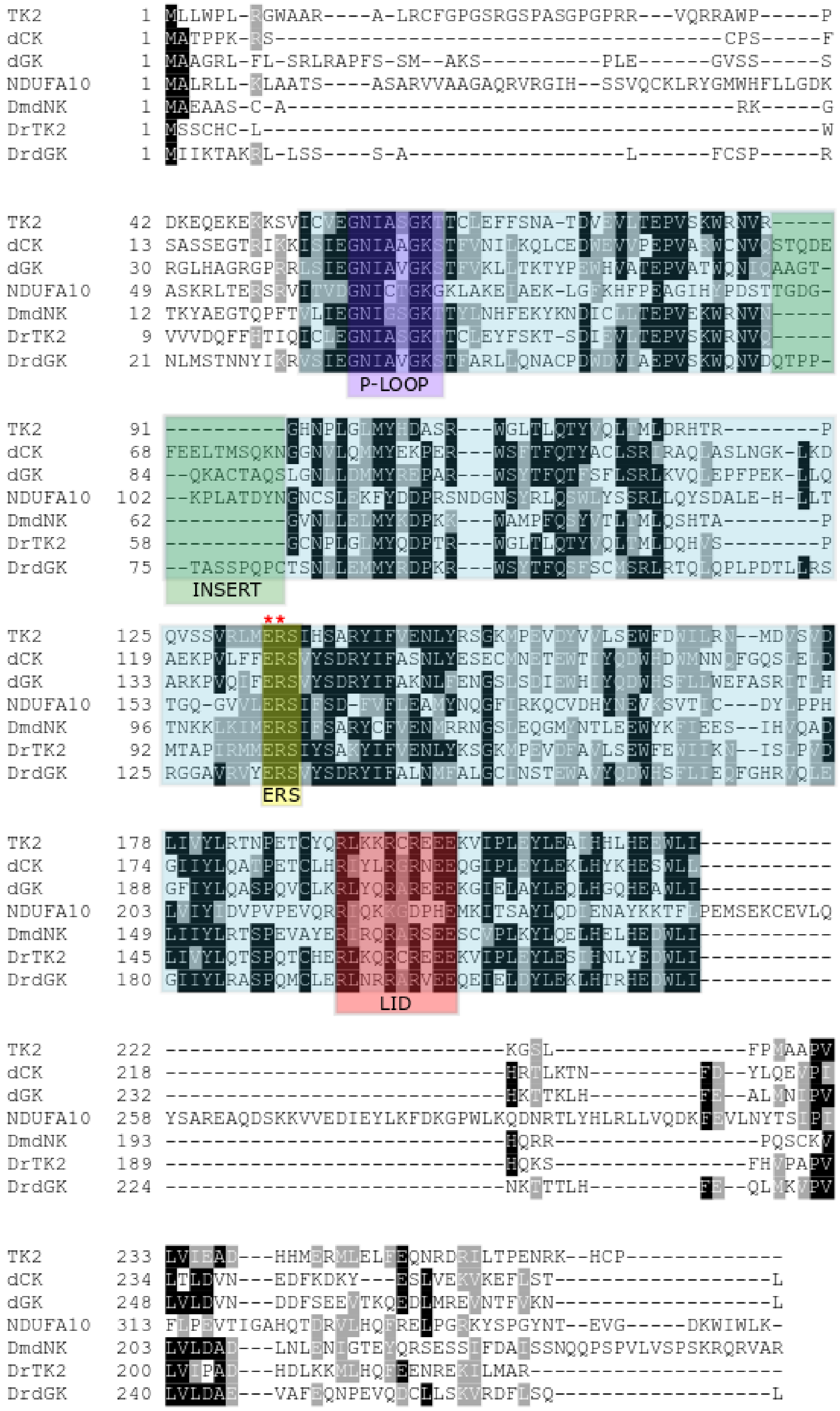

Supplementary Figure 3: Mutations introduced in NDUFA10 deoxyribonucleoside kinase (dNK) domain to disable dGTP binding. Multiple-sequence alignment of

human NDUFA10 and a selection of the dNK protein family members (TK2, *Homo sapiens* thymidine kinase 2; dCK, *Homo sapiens* deoxycytidine kinase; dGK, deoxyguanosine kinase), zebrafish (DmdNK, *Danio rerio* deoxynucleoside kinase; DrTK2, *Danio rerio* thymidine kinase 2) and fruit fly (DmdNK, *Drosophila melanogaster* deoxynucleoside kinase). dNK domain (Pfam PF01712) is shadowed in blue. Color boxes depict conserved motifs (ERS) and regions (Insert; LID (consensus sequence RX_3RXRX_2E) and P-loop (consensus sequence GX_4GKS/T) within the dNK domain. Red asterisks mark altered residues within the ERS motif. FASTA sequences were obtained from Uniprot database (hTK2 (O00142-1); hdCK (P27707-1); hdGK (Q16854-1); hNDUFA10 (O95299-1); DmdNK (Q9XZT6-1); DrTK2 (A0A0R4IP35-1); DrdGK (A5WUY2-1)). Multiple alignment was generated with T-coffee analysis and further formatting with boxshade software.

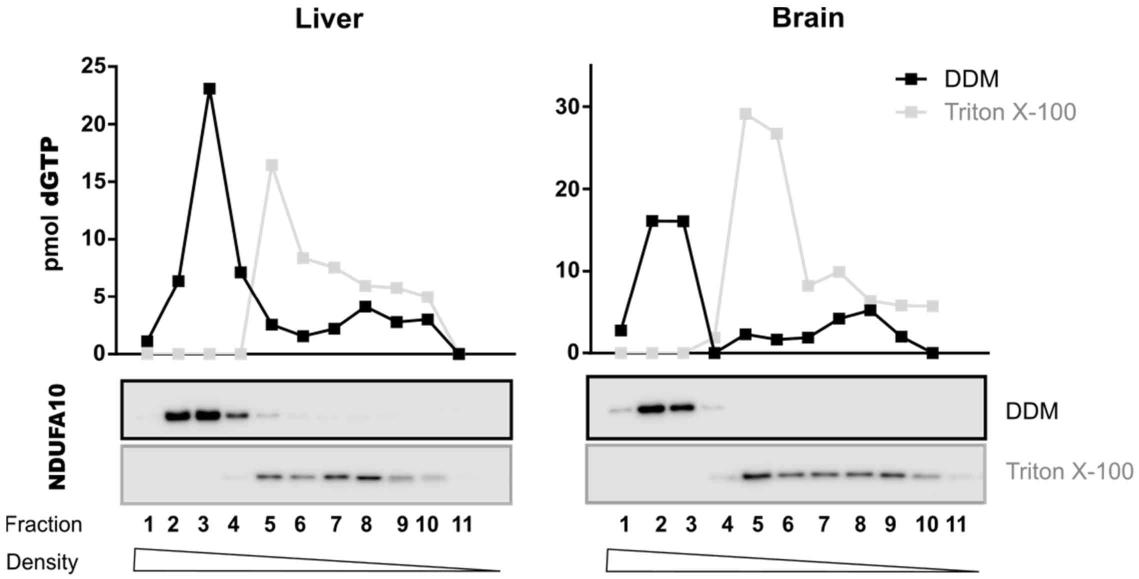

Supplementary Figure 4: Co-migration analysis of dGTP with complex I in sucrose-density gradients with different detergents. 5 mg of mouse liver (left panel) or brain (right panel) mitochondria were lysed in 1% DDM (n-dodecyl β-D-maltoside) (black line and box) or 1% Triton-X-100 (grey line and box), and resolved in 15-37.5% discontinuous sucrose density gradients. Collected fractions were subsequently processed for both dNTP and protein determination. Top graphs show dGTP levels (pmols), and image panels below show complex I migration as analysed by western-blot (anti-NDUFA10 antibody). Data from DDM-gradients is also exposed in Fig 3c.

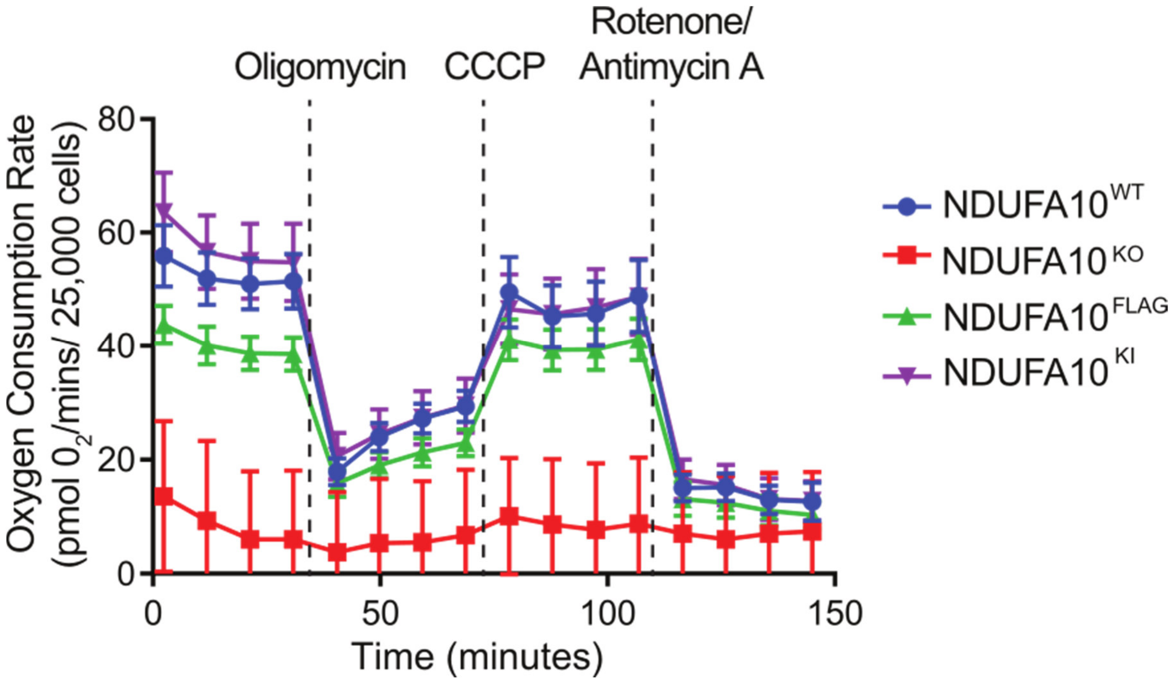

Supplementary Figure 5: Seahorse oxygen consumption assay of NDUFA10 mutant cell lines. Determination of oxygen consumption rate in $NDUFA10^{WT}$, $NDUFA10^{FLAG}$, $NDUFA10^{KO}$, and $NDUFA10^{KI}$ cells. Results show the pmol of O_2 consumed per minute by 25,000 of the cells indicated. The graph shows mean values of 8 independent cultures. Error bars indicate standard deviation.

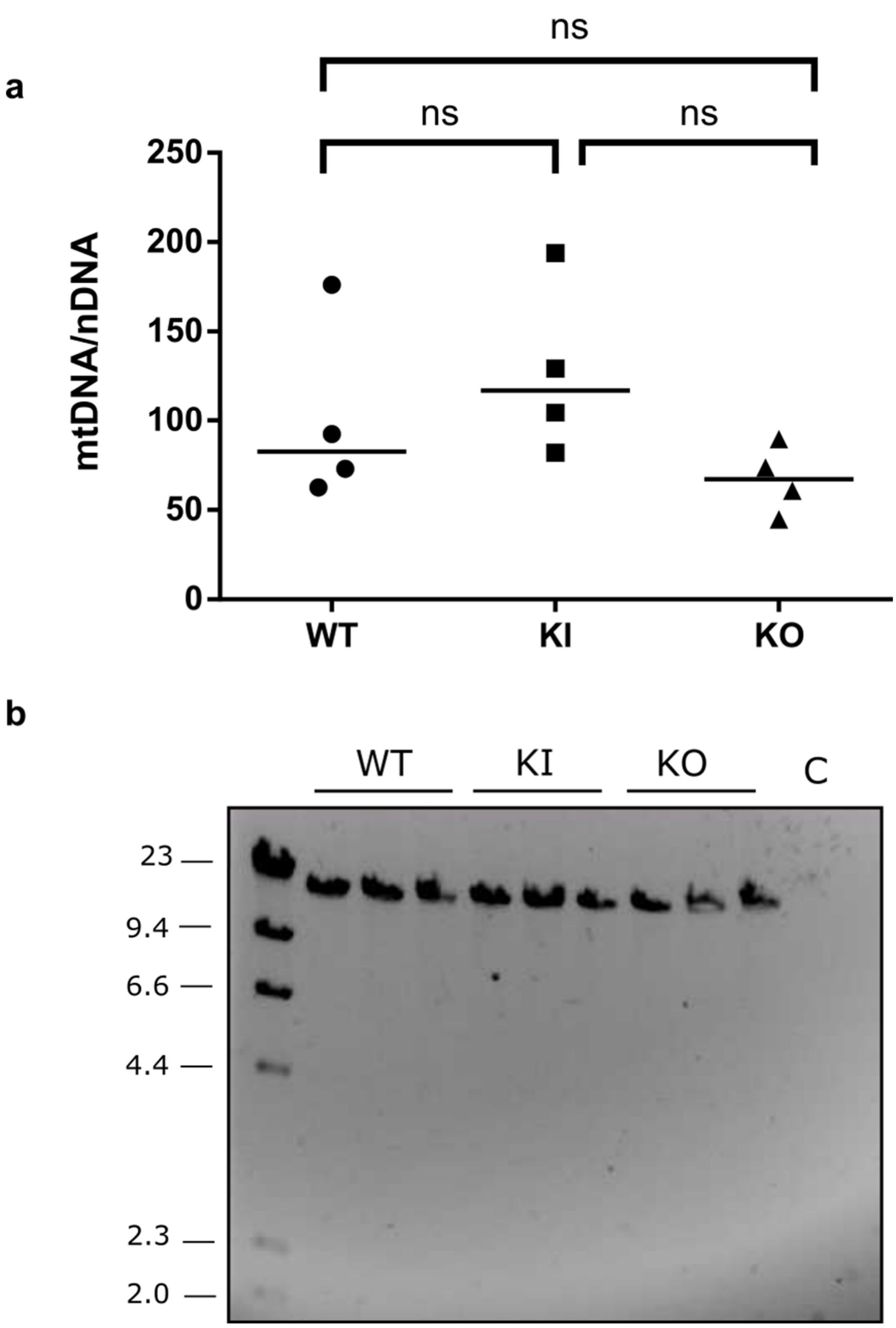

Supplementary Figure 6: MtDNA copy number and deletion analysis in NDUFA10^{KO} and NDFUA10^{KI} cells. a mtDNA and nDNA copy number were determined by quantitative real-time PCR in NDUFA10^{WT} cells (WT), NDUFA10^{KI} (KI), and NDFUA10^{KO} (KO) cell lines. Results indicate the value of mtDNA to nDNA ratio in each cell line. N=4 independent experiments. Scatter plots represent the median (horizontal line). Two-tailed Mann-Whitney U test shows no statistical significance (ns) between groups.

b Amplification of mtDNA molecules by Long PCR in WT, KI and KO cells. C indicates a negative PCR control (water). A normal fragment of nearly 16 Kb corresponding to mtDNA was amplified by Long PCR in DNA samples from 3 independent cultures of WT, KI, and KO cells. Lateral bands indicate molecular weight (kb) of the lambda DNA/HindIII molecular weight marker (Invitrogen).

Supplementary Figure 7: Uncropped western blot images for the indicated figures

Red rectangles depict blotted areas selected for the indicated figures. Blue rectangles indicate samples that were not included in the figures (experimental controls as described). IB: antibodies used for membranes decoration.

Unedited images for Fig. 3b

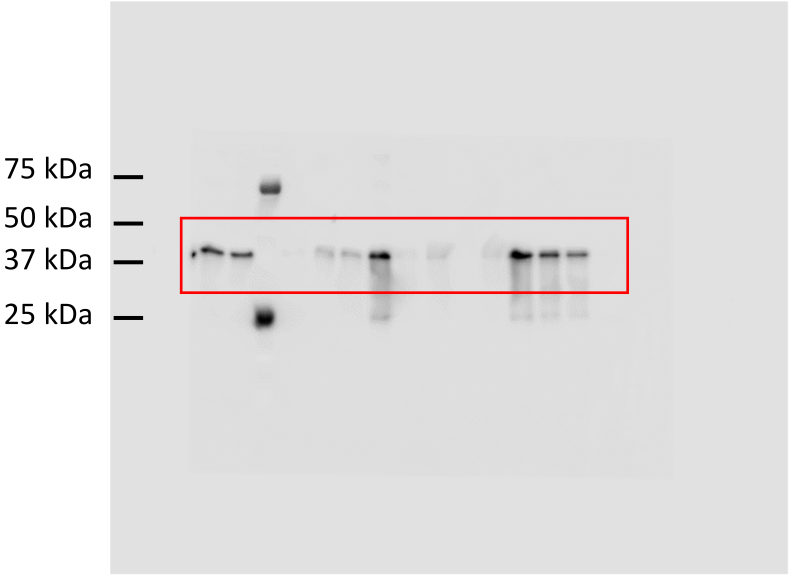

IB: rabbit anti-NDUFA10

liver

Unedited images for Fig. 3c left

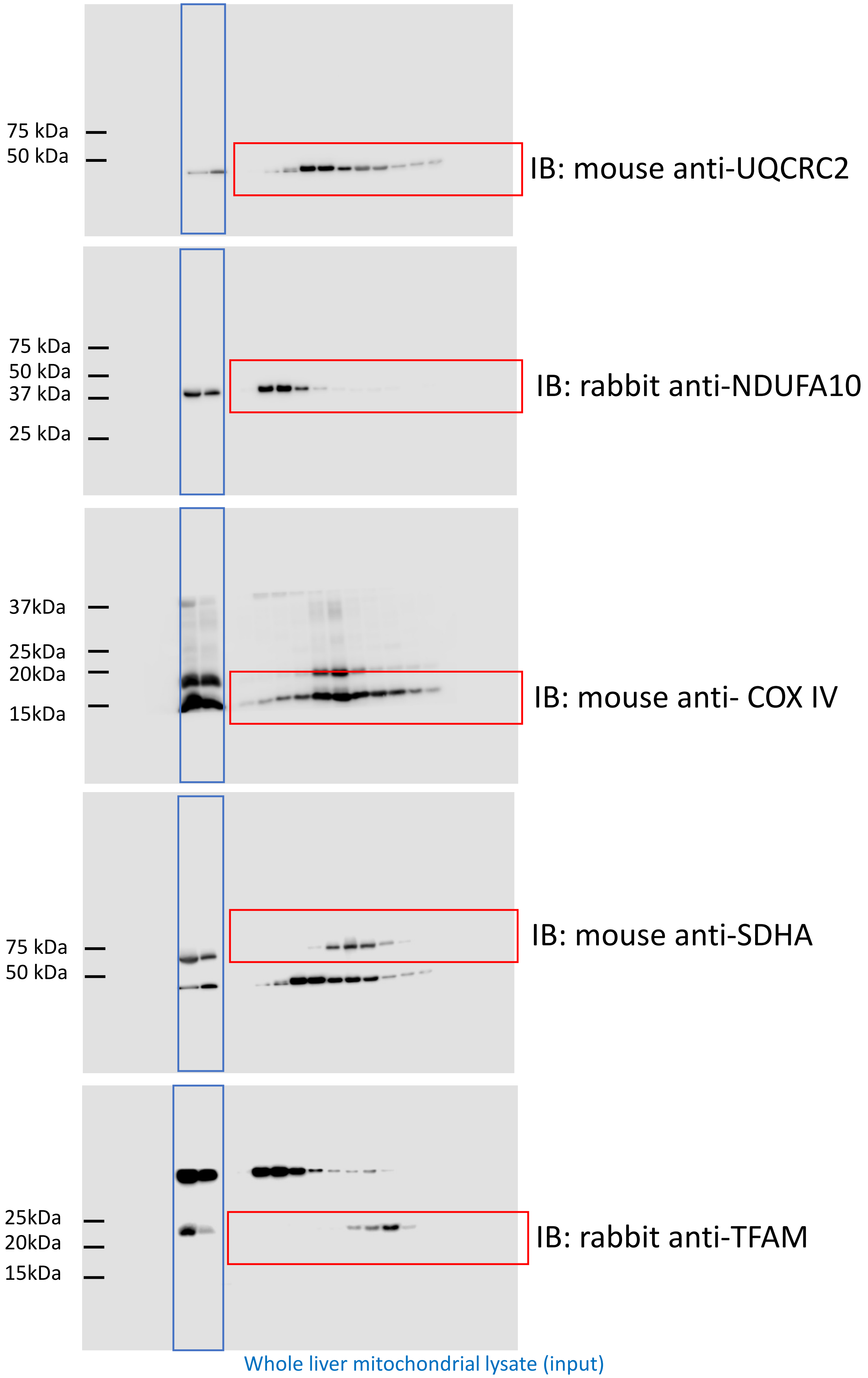

Unedited images for Fig. 3c right

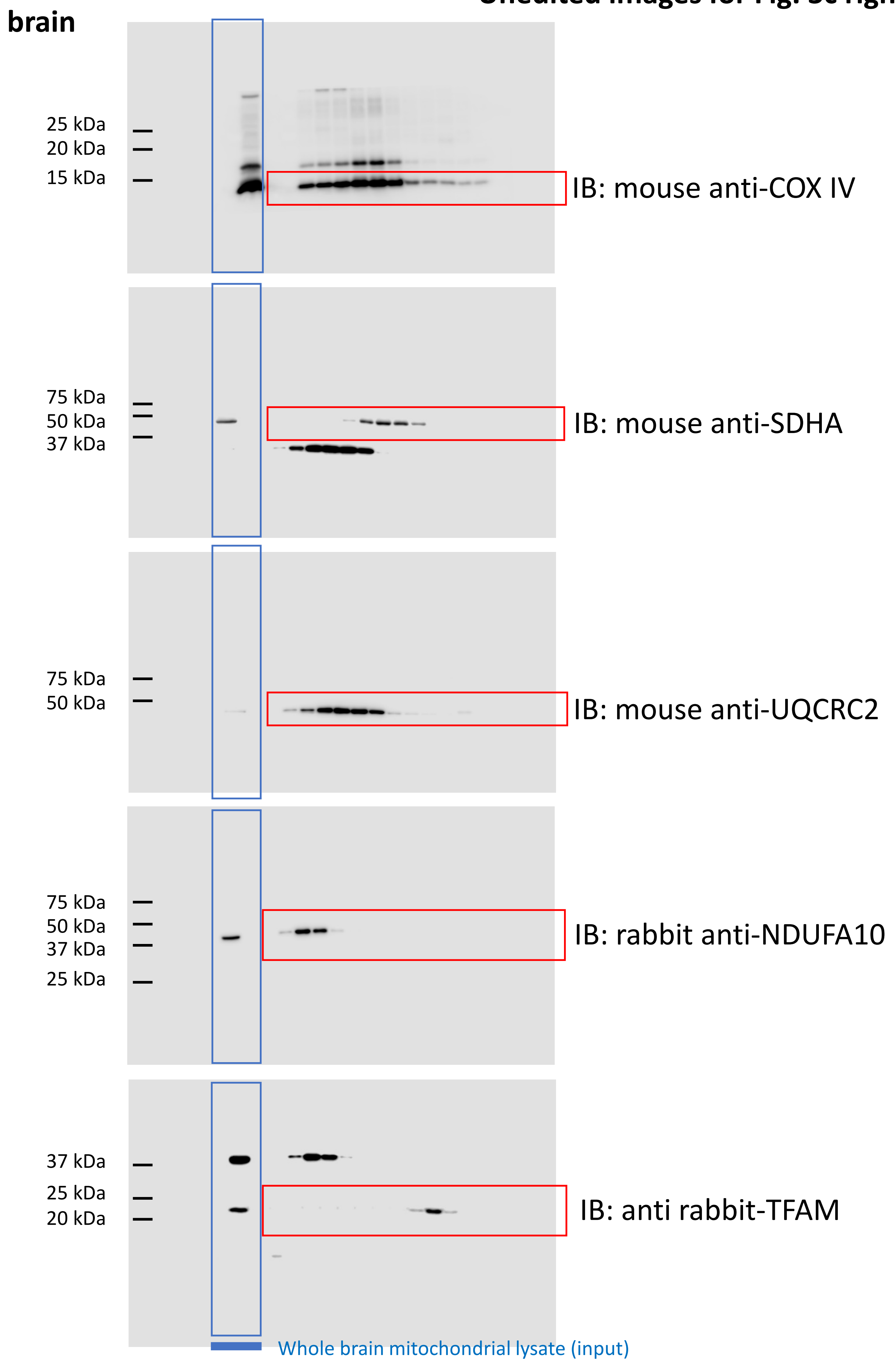

Unedited images for Fig. 3d

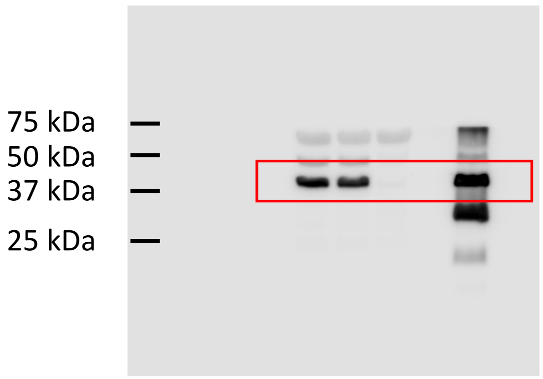

IB: mouse anti-39kDa subunit Complex I

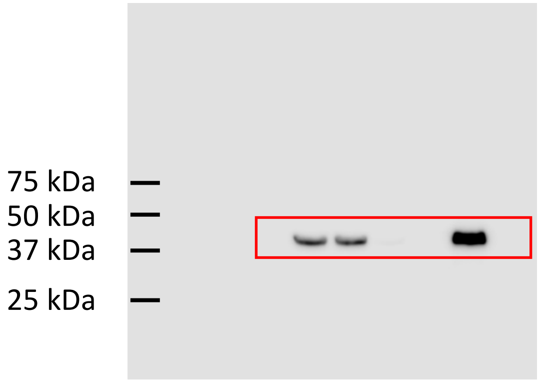

IB: rabbit anti-NDUFA10

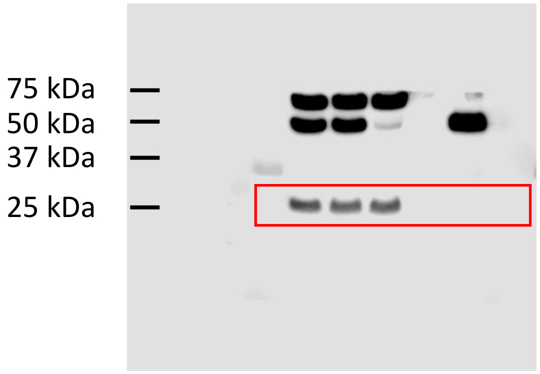

IB: rabbit anti-TFAM

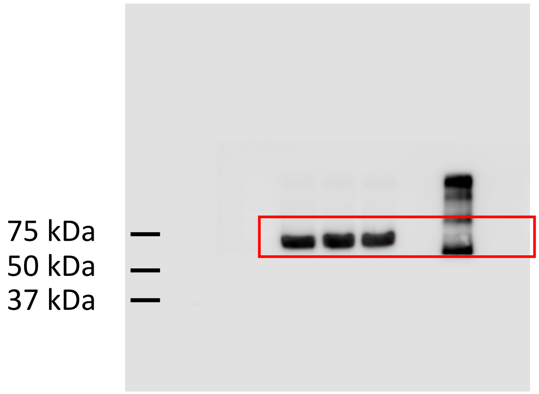

IB: mouse anti-SDHA

Unedited images for Fig. 5a

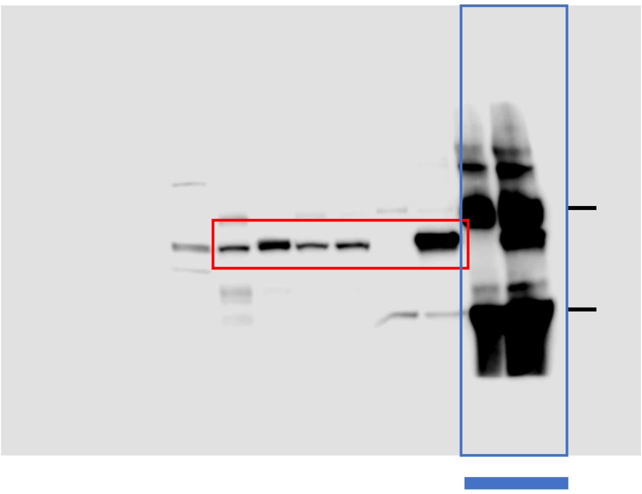

50 kDa (Ig heavy chain)

25 kDa (IG light chain)

Second elution in denaturing conditions

IB: rabbit anti-NDUFA10 (previously blotted with mouse anti-FLAG and anti-mouse secondary antibody)

Unedited images for Fig. 5d

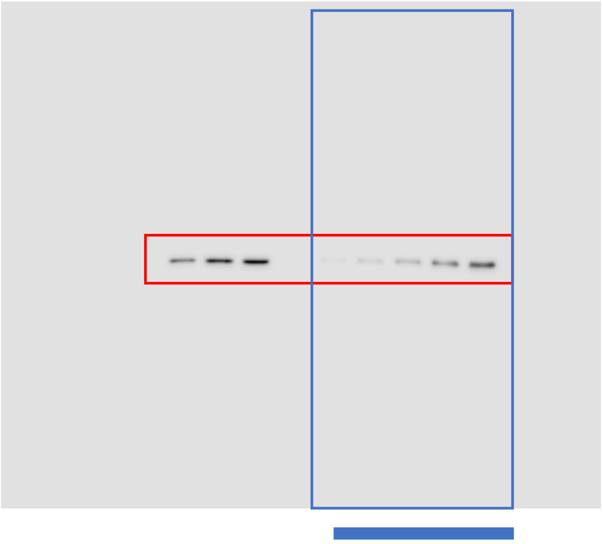

IB: rabbit anti-NDUFA10

Immunopurified NDUFA10FLAG

Unedited images for Fig. 6a

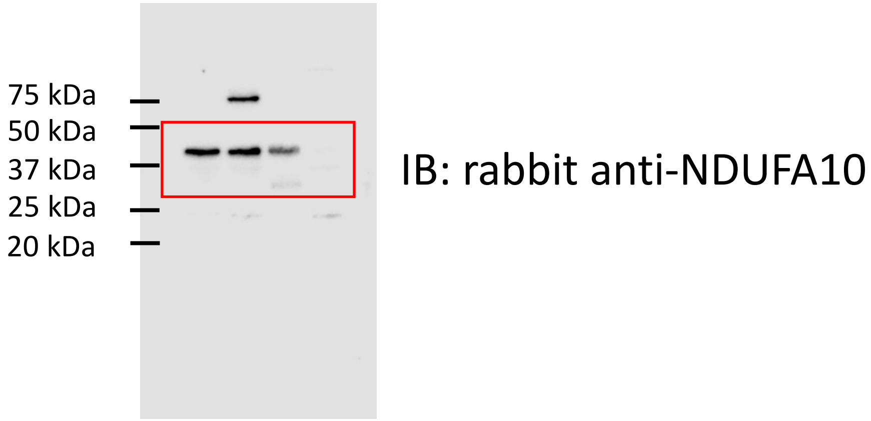

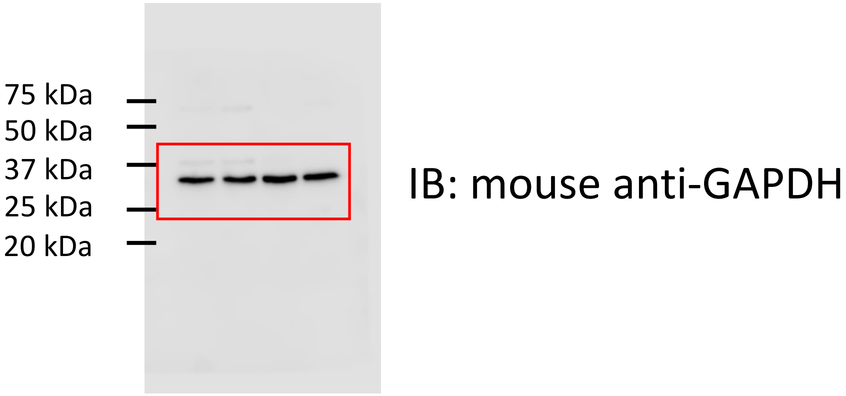

Unedited images for Figure 6d

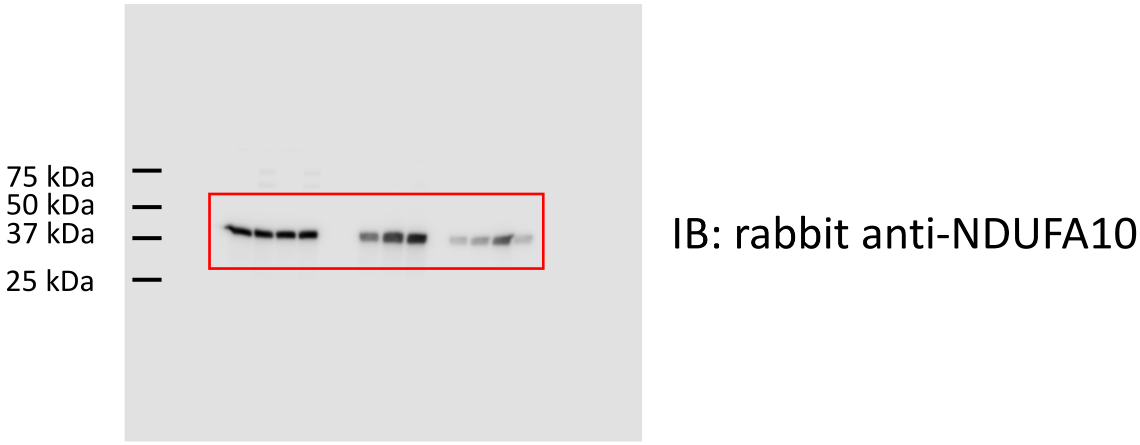

Unedited images for Supplementary Figure 4

brain

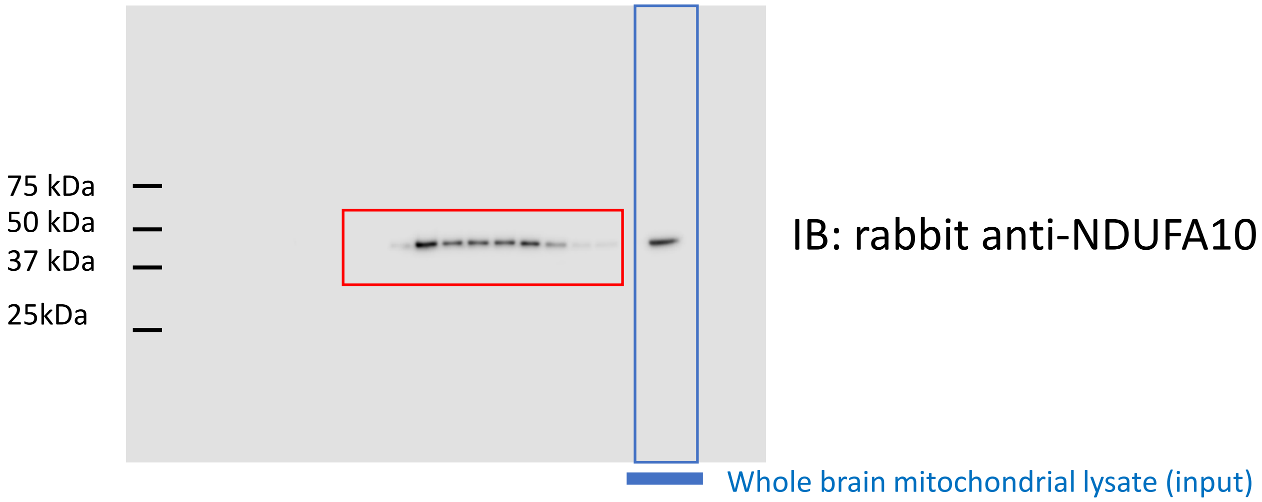

liver

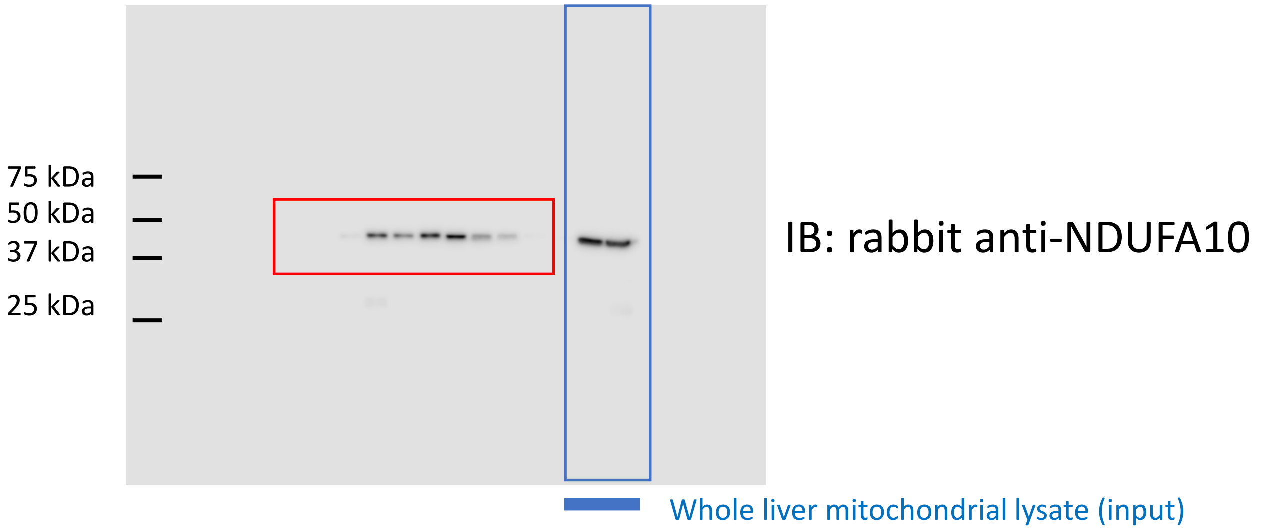

Supplement: Supplementary file 1 — Supplementary information [file 42003_2022_3568_MOESM1_ESM.pdf]
