# Supplementary material for: Most mitochondrial dGTP is tightly bound to respiratory complex I through the NDUFA10 subunit
Source: Commun Biol. 2022 Jun 23;5:620. doi: 10.1038/s42003-022-03568-6 (PMC9226000; doi:10.1038/s42003-022-03568-6)
Supplement: Supplementary file 4 — Reporting summary [file 42003_2022_3568_MOESM4_ESM.pdf]

nature portfolio

Corresponding author(s): Ramon Martí/Yolanda Cámara

Last updated by author(s): May 16, 2022

Reporting Summary

Nature Portfolio wishes to improve the reproducibility of the work that we publish. This form provides structure for consistency and transparency in reporting. For further information on Nature Portfolio policies, see our Editorial Policies and the Editorial Policy Checklist.

Statistics

For all statistical analyses, confirm that the following items are present in the figure legend, table legend, main text, or Methods section.

n/a	Confirmed	
☐	☒	The exact sample size (*n*) for each experimental group/condition, given as a discrete number and unit of measurement
☐	☒	A statement on whether measurements were taken from distinct samples or whether the same sample was measured repeatedly
☐	☒	The statistical test(s) used AND whether they are one- or two-sided<br>*Only common tests should be described solely by name; describe more complex techniques in the Methods section.*
☒	☐	A description of all covariates tested
☒	☐	A description of any assumptions or corrections, such as tests of normality and adjustment for multiple comparisons
☐	☒	A full description of the statistical parameters including central tendency (e.g. means) or other basic estimates (e.g. regression coefficient) AND variation (e.g. standard deviation) or associated estimates of uncertainty (e.g. confidence intervals)
☐	☒	For null hypothesis testing, the test statistic (e.g. *F*, *t*, *r*) with confidence intervals, effect sizes, degrees of freedom and *P* value noted<br>*Give P values as exact values whenever suitable.*
☒	☐	For Bayesian analysis, information on the choice of priors and Markov chain Monte Carlo settings
☒	☐	For hierarchical and complex designs, identification of the appropriate level for tests and full reporting of outcomes
☒	☐	Estimates of effect sizes (e.g. Cohen's *d*, Pearson's *r*), indicating how they were calculated

Our web collection on statistics for biologists contains articles on many of the points above.

Software and code

Policy information about availability of computer code

Data collection	Microbeta 2 (Perkin Elmer) was used for detection of tritiated dNTPs quantification<br>Bioluminiscence was captured with an Odyssey® XF apparatus<br>Absorbance was determined with a sUV-2401PC UV-VIS recording spectrophotometer (Shimadzu Corporation)<br>LC-MS/MS for peptide identification was performed on an LTQ-Orbitrap Velos mass spectrometer (Thermo Scientfic).<br>Oxygen consumption rates were obtained with a Seahorse Bioscience XFe-96 Analyzer<br>qPCR data was generated in a ABI PRISM™ 7900HT (Applied Biossytems) real-time PCR apparatus
Data analysis	PDB and EMDB databases, GRADE, COOT and PyMOL software were used for structural analyses<br>GraphPad Prism 6 software was used for statistical analyses<br>T-coffee software and the Boxshade tool for protein sequence analyses<br>Wave2.6.0 and Prism (v8.0.2, GraphPad) software packages were used for Seahorse data analyses<br>Protein levels were quantified with Image Studio Lite Ver 5.2 software.<br>qPCR analyses were performed using the SDS 2.4 software (Applied Biosystems)<br>Mass spectrometer was controlled with Xcalibur software package, version 2.2.0 (Thermo Fisher Scientific)<br>ProteinScape software (Bruker Daltonics) and Mascot (Matrix Science) were used for peptide identification following LC-MS/MS.

For manuscripts utilizing custom algorithms or software that are central to the research but not yet described in published literature, software must be made available to editors and reviewers. We strongly encourage code deposition in a community repository (e.g. GitHub). See the Nature Portfolio guidelines for submitting code & software for further information.

Data

Policy information about availability of data

All manuscripts must include a data availability statement. This statement should provide the following information, where applicable:

- Accession codes, unique identifiers, or web links for publicly available datasets
- A description of any restrictions on data availability
- For clinical datasets or third party data, please ensure that the statement adheres to our policy

All raw data underlying the graphs and charts presented in the main and supplementary figures are present in the Supplementary Data files 1 and 2, respectively. Uncropped images for western-blots are included in Supplementary Figure 7. Radiochemicals, antibodies and other materials used in the study are listed in Supplementary Tables 2-7. Structural data use for modeling NDUFA10 binding to dGTP are available at https://www.rcsb.org (10.2210/pdb6ZKA/pdb; 10.2210/pdb6g2j/pdb). The mass spectrometry datasets generated during the dGTP pull-down studies have been deposited to the ProteomeXchange Consortium via the PRIDE partner repository with the dataset identifier PXD033900. All other data are available from the corresponding authors on reasonable request.

Field-specific reporting

Please select the one below that is the best fit for your research. If you are not sure, read the appropriate sections before making your selection.

☒ Life sciences ☐ Behavioural & social sciences ☐ Ecological, evolutionary & environmental sciences

For a reference copy of the document with all sections, see nature.com/documents/nr-reporting-summary-flat.pdf

Life sciences study design

All studies must disclose on these points even when the disclosure is negative.

Sample size	A minimum sample size of 4 was established based on our previous observation that, for virtually all variables studied in this work, we were able to detect statistically significant differences with non-parametric methods.
Data exclusions	Occasionally, some data was excluded if technical interference was objectively identified
Replication	All quantifiable results include samples tested/obtained in different independent experiments/individuals. For non-quantifiable obrservations, all results were reproduced at least in one additional independent experiment.
Randomization	Randomization was not required in our experimental setting, because all experients included all groups to be compared.
Blinding	Blinding was not possible since genetically-modified cells or specific treatments needed to be identifiable usually when starting the experiment. Biased interpretation was prevented by review of raw data by at least two-independent investigators.

Reporting for specific materials, systems and methods

We require information from authors about some types of materials, experimental systems and methods used in many studies. Here, indicate whether each material, system or method listed is relevant to your study. If you are not sure if a list item applies to your research, read the appropriate section before selecting a response.

Materials & experimental systems

n/a	Involved in the study	
☐	☒	Antibodies
☐	☒	Eukaryotic cell lines
☒	☐	Palaeontology and archaeology
☐	☒	Animals and other organisms
☒	☐	Human research participants
☒	☐	Clinical data
☒	☐	Dual use research of concern

Methods

n/a	Involved in the study	
☒	☐	ChIP-seq
☒	☐	Flow cytometry
☒	☐	MRI-based neuroimaging

Antibodies

Antibodies used	Anti-NDUFA10 antibody (GeneTex # GTX114572); Anti-Core II Complex III (Molecular Probes #A11143); Anti-COX IV (Abcam #ab16056); Anti-SDHA70 (Abcam #ab14715); Anti-VDAC (Abcam #ab15895); Anti-TFAM (GeneTex #GTX103231); Anti-39kDa subunit Complex I (Molecular Probes #A21344); Anti-FLAG M2 antibody (Sigma-Aldrich #F3165); Anti-GAPDH (Origene #TA802519)
Validation	Validations were based on data form manufacturers:<br>Anti-NDUFA10 antibody (GeneTex # GTX114572): negative for NDUFA10 knock-out human cells in the manuscript; https://

www.genetex.com/Product/Detail/NDUFA10-antibody/GTX114572
Anti-Core II Complex III (Invitrogen #A-11143) and Anti-39kDa subunit Complex I (Invitrogen #A21344): http://tools.thermofisher.com/content/sfs/manuals/mp06401.pdf
Anti-COX IV (Abcam #ab16056): https://www.abcam.com/cox-iv-antibody-mitochondrial-loading-control-ab16056.html
Anti-SDHA70 (Abcam #14715): https://www.abcam.com/sdha-antibody-2e3gc12fb2ae2-ab14715.html
Anti-VDAC (Abcam #15895): https://www.abcam.com/vdac1porin-antibody-mitochondrial-loading-control-ab15895.html
Anti-TFAM (GeneTex #GTX103231): https://www.genetex.com/Product/Detail/mtTFA-antibody/GTX103231
Anti-39kDa subunit Complex I (Molecular Probes #A21344) http://tools.thermofisher.com/content/sfs/manuals/mp06401.pdf
Anti-FLAG M2 antibody (Sigma-Aldrich #F3165): positive for human cells transfected with Flag-tagged NDUFA10 in the manuscript; https://www.sigmaaldrich.com/ES/es/product/sigma/f3165?gclid=CjwKCAiAjoeRBhAJEiwAYY3nDHiIa0MX4tBD0pN6k-I5TKp76uHGoewqy1k0_H_dn9EYHeI_OmCwHBoCGD8QAvD_BwE
Anti-GAPDH (Origene #TA802519): https://www.origene.com/products/antibodies/primary-antibodies/loading-control-antibodies/gapdh-loading-control

Eukaryotic cell lines

Policy information about cell lines

Cell line source(s)
293HEK cells were originally purchased from the ATCC and modified in Dr. Ryan's lab as described in Stroud et al, 2016
HeLa cells were obtained from collaborators and transformed with lentivirus for NDUFA10-FLAG expression and purification

Authentication
None of the cell lines in the study were later authenticated

Mycoplasma contamination
Cell lines were not tested for Mycoplasma contamination

Commonly misidentified lines
(See ICLAC register)
None

Animals and other organisms

Policy information about studies involving animals; ARRIVE guidelines recommended for reporting animal research

Laboratory animals
Male C57Bl6 mice (acquired from Charles River Laboratories) from 8-20 week of age were used

Wild animals
n/a

Field-collected samples
n/a

Ethics oversight
The protocol was approved by the Ethics Committee for Animal Experimentation of the Vall d'Hebron Research Institute (Permit Number: 73/19) and by the Generalitat de Catalunya

Note that full information on the approval of the study protocol must also be provided in the manuscript.
