# Supplementary material for: Most mitochondrial dGTP is tightly bound to respiratory complex I through the NDUFA10 subunit
Source: Commun Biol. 2022 Jun 23;5:620. doi: 10.1038/s42003-022-03568-6 (PMC9226000; doi:10.1038/s42003-022-03568-6)
Supplement: Supplementary file 6 — Description of Additional Supplementary Files [file 42003_2022_3568_MOESM6_ESM.pdf]

Description of Additional Supplementary Files

File Name: Supplementary Data 1

Description: Source data for graphs in Main manuscript figures

File Name: Supplementary Data 2

Description: Source data for graphs in Supplementary figures
